# The Somatostatin Analogue Octreotide Inhibits Growth of Small Intestine Neuroendocrine Tumour Cells

**DOI:** 10.1371/journal.pone.0048411

**Published:** 2012-10-31

**Authors:** Su-Chen Li, Cécile Martijn, Tao Cui, Ahmed Essaghir, Raúl M. Luque, Jean-Baptiste Demoulin, Justo P. Castaño, Kjell Öberg, Valeria Giandomenico

**Affiliations:** 1 Department of Medical Sciences, Endocrine Oncology, Science for Life Laboratory, Uppsala University, Uppsala, Sweden; 2 Department of Surgical Sciences, Anaesthesiology & Intensive Care, Science for Life Laboratory, Uppsala University, Uppsala, Sweden; 3 Université Catholique de Louvain, de Duve Institute, Brussels, Belgium; 4 Department of Cell Biology, Physiology, and Immunology, Instituto Maimónides de Investigación Biomédica (IMIBIC), Hospital Universitario Reina Sofia, University of Cordoba, and CIBER Fisiopatología de la Obesidad y Nutrición (CIBERobn), Cordoba, Spain; 5 Centre of Excellence for Endocrine Tumours, Uppsala University Hospital, Uppsala, Sweden; University of Santiago de Compostela School of Medicine - CIMUS, Spain

## Abstract

Octreotide is a widely used synthetic somatostatin analogue that significantly improves the management of neuroendocrine tumours (NETs). Octreotide acts through somatostatin receptors (*SSTRs*). However, the molecular mechanisms leading to successful disease control or symptom management, especially when *SSTRs* levels are low, are largely unknown. We provide novel insights into how octreotide controls NET cells. CNDT2.5 cells were treated from 1 day up to 16 months with octreotide and then were profiled using Affymetrix microarray analysis. Quantitative real-time PCR and western blot analyses were used to validate microarray profiling *in silico* data. WST-1 cell proliferation assay was applied to evaluate cell growth of CNDT2.5 cells in the presence or absence of 1 µM octreotide at different time points. Moreover, laser capture microdissected tumour cells and paraffin embedded tissue slides from SI-NETs at different stages of disease were used to identify transcriptional and translational expression. Microarrays analyses did not reveal relevant changes in *SSTR* expression levels. Unexpectedly, six novel genes were found to be upregulated by octreotide: annexin A1 (*ANXA1*), rho GTPase-activating protein 18 (*ARHGAP18*), epithelial membrane protein 1 (*EMP1*), growth/differentiation factor 15 (*GDF15*), TGF-beta type II receptor (*TGFBR2*) and tumour necrosis factor (ligand) superfamily member 15 (*TNFSF15*). Furthermore, these novel genes were expressed in tumour tissues at transcript and protein levels. We suggest that octreotide may use a potential novel framework to exert its beneficial effect as a drug and to convey its action on neuroendocrine cells. Thus, six novel genes may regulate cell growth and differentiation in normal and tumour neuroendocrine cells and have a role in a novel octreotide mechanism system.

## Introduction

Neuroendocrine tumours (NETs) are rare cancers that can affect different parts of the body. Gastrointestinal (GI) NETs originate from enterochromaffin (EC) cells, which are sparse neuroendocrine cells disseminated throughout the GI tract [1], [2]. A subgroup of GI-NETs are small intestine NETs (SI-NETs). A recent and detailed WHO classification for GI-NETs is available in a recent paper [3].

The primary SI-NET is often small and asymptomatic. Therefore, diagnosis and treatment may be delayed by several years, during which metastases can form. Radical surgical resection alone can be curative in patients with early stage disease. However, most patients present advanced disease at the time of diagnosis [4]. Thus, the current treatments of metastasized SI-NETs aim at controlling tumour growth and hormonal secretion by using mainly somatostatin analogues (SSAs), interferon alpha and more recently everolimus [5], [6], [7], [8].

Several earlier studies reported that SSAs exert their effects by binding to SSTRs. Five genes *SSTR1-SSTR5* encode five receptors named SSTR1 to SSTR5 respectively. They signal and activate cellular and molecular mechanisms leading to either successful therapy or acquired resistance to the drugs [9], [10]. Octreotide (Sandostatin®) was the first SSA commercially available. In addition, it has high affinity for SSTR2 and moderate affinity for SSTR3 and SSTR5 [11]. SSAs have long been used to treat NETs. Somatostatin is a natural growth-hormone-releasing inhibiting factor produced in the hypothalamus, which exerts its effects through high-affinity to somatostatin receptors (SSTRs). These receptors are G coupled protein receptors and elicit cellular responses through second-messenger systems [12]. The introduction of octreotide, the first SSA in 1987 and later novel SSAs resulted in NETs symptom management. However, their capacity to inhibit tumour growth has been seriously debated for a long time. The first performed placebo-controlled, double-blinded, phase IIIB study in patients with well-differentiated metastatic midgut NETs addressed the hypothesis that octreotide-lanreotide (LAR) prolongs time to tumour progression and survival. The study concluded that octreotide-LAR significantly lengthens time to tumour progression compared to placebo in patients with functionally active and inactive metastatic midgut NETs [13]. The conclusions of the study have been debated due to its design and the number of patients included [14], [15]. Nevertheless, clinical results confirmed that octreotide and lanreotide provide in addition to their suppression of carcinoid syndrome, antitumour benefits in terms of tumour cell growth control [13]. In summary, somatostatin analogs (including octreotide and lanreotide) have been indicated for the relief of the symptoms of flushing, diarrhea, and wheezing associated with secretory neuroendocrine tumors (NETs). Recently, it has been suggested that somatostatin analogs may provide direct and indirect antitumor effects in secretory and nonsecretory NETs in addition to symptom control in secretory NETs and more findings have been explored about the octreotide anti-angiogenic role. However, many aspects of octreotide control on tumour growth are still largely unclear [16], [17], [18].

**Figure 1:**
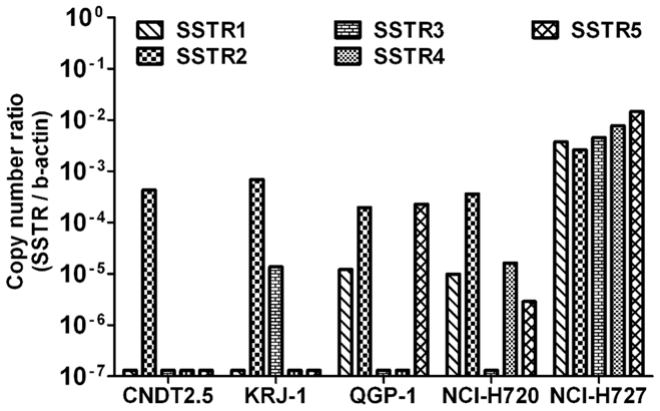
*SSTR1, SSTR2, SSTR3, SSTR4 and SSTR5* gene expression analysis. *SSTR1-5* gene expression analysis was performed by QRT-PCR on total RNA from five human NET cell lines: CNDT2.5, KRJ-1, QGP-1, NCI-H720 and NCI-H727. The absolute mRNA copy numbers are adjusted by *β-actin* mRNA copy number. Results were plotted using the 2^−ΔΔCt^ method with *β-actin* expression (set to 1) from each individual sample as endogenous reference.

**Figure 2:**
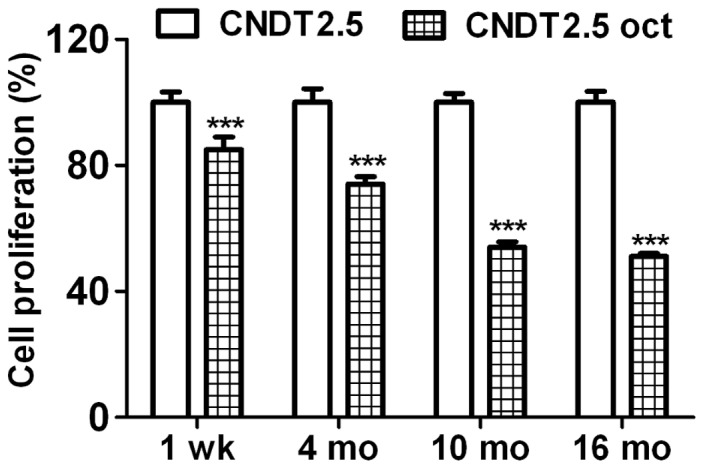
CNDT2.5 cells growth in the presence of 1 µM octreotide was kinetically evaluated. Cells were cultured in the absence or presence of 1 µM octreotide (oct). WST-1 assay was used to evaluate cell growth. Cell proliferation ratio for each time point was converted to a percentage of the mean value relative to CNDT2.5 cells growth, set to 100%. Plotted results are means ± SD from triplicate wells. Significance was calculated by using Two-Way ANOVA followed by Bonferroni test; comparing with untreated CNDT2.5 cells. *** = *p*<0.001.

For instance, it is still not known where octreotide can reduce cell growth in absence of a SSTR pathway. However, the mechanism that triggers resistance to octreotide has not been identified. Recently, it has been reported that long-term treatments with SSAs, such as octreotide, may switch NET cells to be static in growth activating apoptosis or cell cycle arrest at G_1_ [19]. However, clinical transposition of the results produced *in vitro* is difficult [20]. This finding suggests and supports the idea that SSAs may use more complex alternative mechanisms, which have not been fully addressed and deserve further investigation.

Although the number of cell lines available from SI-NETs is limited and they cannot fully mimic a malignancy, they can still broaden our understanding of NET cell biology and serve as tools for development of novel therapies [21], [22], [23], [24], [25], [26]. The present study started by testing five human NET cell lines, CNDT2.5 [27] and KRJ-1 [28], established from SI-NETs, QGP-1 from a pancreatic NET [29]; and NCI-H720 and NCI-H727 from lung carcinoids. The molecular basis underlying octreotide growth and differentiation control of neuroendocrine cells is elusive and would surely benefit from the establishment of a novel representative *in vitro* model.

**Table 1:** Gene ontology of 25 selected genes from microarray analyses.

Symbol	Description	Molecular function	Biological process	Oct/control
ABHD5	abhydrolase domain 5	protein binding	lipid metabolism	1.2
ADAMTSL1	ADAMTS-like 1	metallopeptidase activity	–	1.5
**ANXA1**	**annexin A1**	phospholipase A2 inhibitor	cell proliferation	1.3
**ARHGAP18**	**Rho GTPase activating protein 18**	GTPase activator activity	signal transduction	1.4
ASS1	argininosuccinate synthetase 1	argininosuccinate synthase activity	urea cycle	1.5
C1orf110	chromosome 1 open reading frame 110	–	–	1.3
CDH13	cadherin 13	calcium binding	cell proliferation	1.3
**EMP1**	**epithelial membrane protein 1**	–	cell proliferation	1.4
ERRFI	ERBB receptor feedback inhibitor 1	Rho GTPase activator activity	signal transduction	1.2
FLNB	filamin B	protein binding	development	1.1
**GDF15**	**growth differentiation factor 15**	growth factor activity	signal transduction	1.4
HBEGF	heparin-binding EGF-like growth factor	epodermal growth factor receptor binding	signal transduction	−1.2
LAMA1	laminin, alpha 1	receptor binding	signal transduction	1.3
MAP1LC3C	microtubule-associated protein 1 light chain 3	–	autophagy	−1.3
MICAL2	microtubule associated monoxygenase	metal ion binding	metabolism	1.2
MYEOV	myeloma overexpressed	–	cell migration	1.3
NEDD4L	neural precursor cell, down-regulated 4-like	sodium channel regulator activity	sodium ion transport	1.3
PDP1	pyruvate dehyrogenase phosphatase 1	protein serine/threonine phosphatase activity	protein amino acid dephosphorylation	1.2
PLAU	plasminogen activator, urokinase	serine-type endopeptidase activity	signal transduction	1.3
RGS5	regulator of G-protein signaling 5	GTPase activator activity	G-protein coupled receptor protein signaling pathway	1.0
RUNX2	runt-related transcription factor 2	transcription factor activity	negative regulation of transcription	1.2
S100A10	S100 calcium binding protein A10	receptor binding	signal transduction	1.2
SQSTM1	sequestosome 1	SH2 domain binding	signaling cascade	1.2
**TGFBR2**	**transforming growth factor, β receptor II**	protein serine/threonine phosphatase activity	common-partner SMAD protein phosphorylation	1.3
**TNFSF15**	**tumor necrosis factor** **superfamily member 15**	tumor necrosis factor receptor binding	signal transduction	1.5

Log_2_ ratio expression between octreotide (Oct) treated CNDT2.5 cells and untreated CNDT2.5 cells (control). Bolded genes were further analyzed.

**Figure 3:**
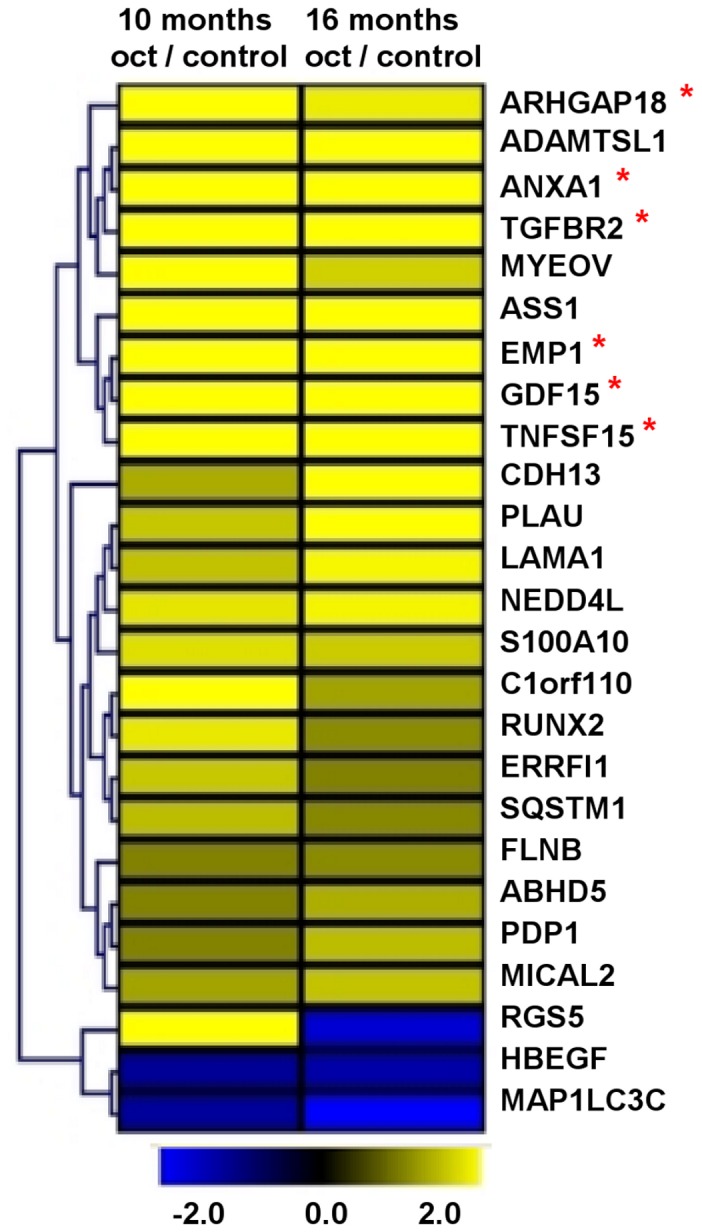
Gene expression of CNDT2.5 cells and 1 µM octreotide treated cells. Microarray analysis detected 25 differentially expressed genes in octreotide treated CNDT2.5 cells compared to CNDT2.5 untreated cells (control). Upregulated genes are in yellow and downregulated genes are in blue. Genes were clustered according to Euclidian distance, as indicated in the figure. Of the 25 genes, 6 were selected for further analysis and they are indicated by a red asterisk.

Affymetrix microarray analyses and quantitative real time PCR (QRT-PCR) showed that CNDT2.5 has the lowest expression of *SSTR1-5*. This made CNDT2.5 a suitable model *in vitro* for our analysis. CNDT2.5 cells were treated with octreotide for between 1 week and 16 months. Then genome-wide transcript profiling was used to compare treated and untreated cells. Results showed that octreotide had the ability to reduce CNDT2.5 cell growth persistently and, most importantly, revealed the presence of a small group of six genes, previously unrelated to octreotide-induced signaling, which may be involved in cell growth rate reduction and differentiation of neuroendocrine cells.

## Results

### Quantitative Real Time PCR (QRT-PCR) Analysis Shows that CNDT2.5 Express Limited Amount of *SSTRs*

We calculated copy number of the transcripts of the five somatostatin receptors (*SSTRs 1–5*) in five human neuroendocrine cancer cell lines. To investigate the expression of *SSTRs* the copy number of *β-actin* was used as internal control. The results showed a varied expression of *SSTRs* pattern (Figure 1). Based on these data, CNDT2.5 was selected as a suitable model for subsequent studies given their profile and levels of *SSTR* expression.

**Figure 4:**
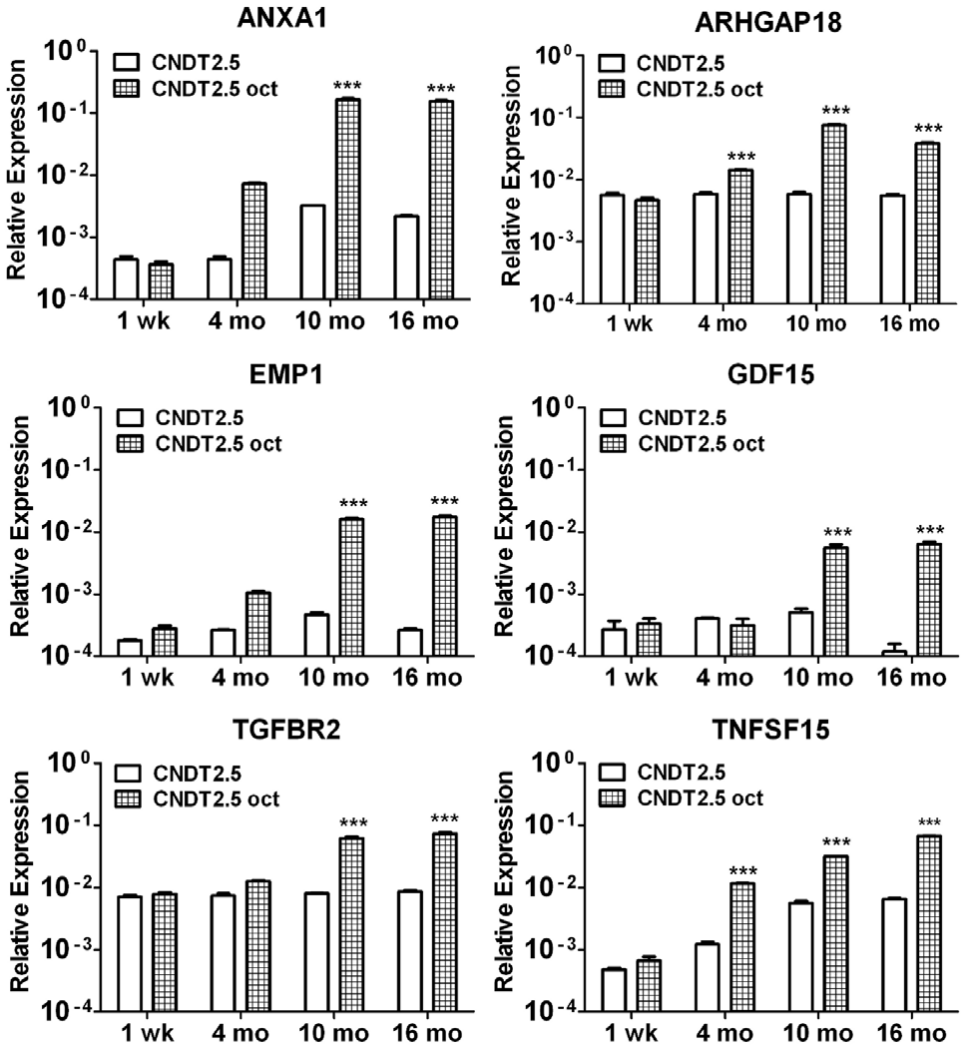
QRT-PCR analysis on CNDT2.5 cells in the absence or presence of 1 µM octreotide. *ANXA1, ARHGAP18, EMP1, GDF15, TGFBR2* and *TNFSF15* were analysed using total RNA at 1 week (wk), 4 months, 10 months and 16 months (mo) of culture by QRT-PCR. Results were plotted using the 2^−ΔΔCt^ method with *β-actin* expression (set to 1) from each individual sample as endogenous reference. Plotted results are means ± SD for triplicate wells. Significance was calculated by Two-Way ANOVA followed by Bonferroni test; comparing with untreated CNDT2.5 cells. *** = *p*<0.001.

**Figure 5:**
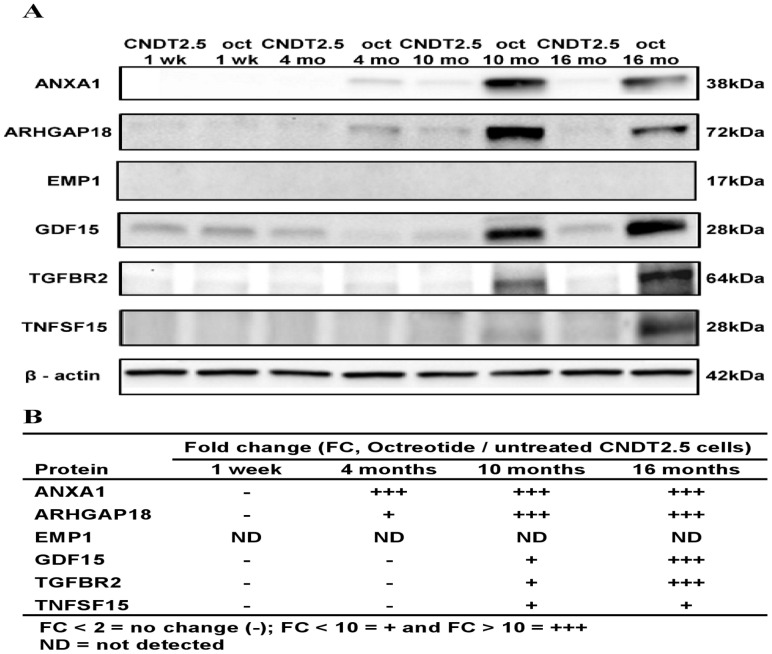
ANXA1, ARHGAP18, EMP1, GDF15, TGFBR2 and TNFSF15 Western blot analysis. CNDT2.5 cells cultured in the absence or presence of 1 µM octreotide were collected at 1 week (wk), 4 months, 10 months and 16 months (mo) to prepare total lysates. Octreotide induces protein expression level of octreotide treated CNDT2.5 cells for 10 and 16 months compared to untreated cells (5A). β-actin was used as endogenous control. Fold changes are illustrated in 5B.

**Table 2:** Small intestine neuroendocrine tumour specimens and analyses.

Patient	Sex/Age^*^	Treatment	Type	Tissue	Analysis
1	M/70	Untreated	Primary	Ileum	QRT-PCR, LCM (FS)
2	F/63	Untreated	Primary	Ileum	QRT-PCR, LCM (FS)
3	F/63	Untreated	Primary	Ileum	QRT-PCR, LCM (FS), IHC (PET)
4	M/54	Untreated	Primary	Ileum	IHC (PET)
5	F/59	Untreated	Primary	Ileum	IHC (PET)
6	F/68	Untreated	Metastases	Liver	IHC (PET)
7	M/60	Untreated	Metastases	Liver	IHC (PET)
8	F/71	Untreated	Metastases	Liver	IHC (PET)
9	F/57	SSA+IFN	Primary	Ileum	IHC (PET)
10	F/58	SSA+IFN	Primary	Ileum	IHC (PET)
11	F/52	SSA+IFN	Primary	Ileum	IHC (PET)
12	F/67	SSA+IFN	Metastases	Liver	QRT-PCR, LCM (FS), IHC (PET)
13	F/65	SSA+IFN	Metastases	Liver	QRT-PCR, LCM (FS)
14	F/76	SSA+IFN	Metastases	Liver	QRT-PCR, LCM (FS)
15	M/61	SSA	Metastases	Liver	IHC (PET)
16	F/44	SSA+IFN	Metastases	Mesentery	IHC (PET)

Age at the time of operation (Age*); Somatostatin Analogues (SSA); Interferon α (IFN).

Laser Capture Microdissection (LCM); Frozen Specimen (FS); Paraffin-Embedded Tissue (PET).

**Table 3:** Result of Immunohistochemistry on paraffin embedded SI-NET specimens.

Protein	P		L		M	Positive Staining
	UT	T	UT	T	T	
ANXA1	3/3–	3/3–	3/3–	2/2–	1/1–	0/12
ARHGAP18	2/3+++	2/3++	1/3+++	2/2+++	1/1++	12/12
	1/3++	1/3+	2/3++			
EMP1	1/3+++	3/3+++	3/3+++	1/2+++	1/1+++	12/12
	2/3++			1/2++		
GDF15	1/3+++	3/3++	1/3++	1/2+++	1/1++	12/12
	1/3++		2/3+	1/2+		
	1/3+					
TGFBR2	2/3+	3/3+	1/3+	1/2+	1/1++	8/12
	1/3–		2/3–	1/2–		
TNFSF15	1/3+++	2/3+++	1/3+++	1/2+	1/1+	11/12
	1/3++	1/3++	2/3+	1/2–		
	1/3+					

Primary tumour (P); Liver metastases (L); Mesentery metastases (M); Untreated (UT); Treated (T).

Intensity in >50% of tumour cells: +++ strong, ++ moderate, + weak, – negative.

**Figure 6:**
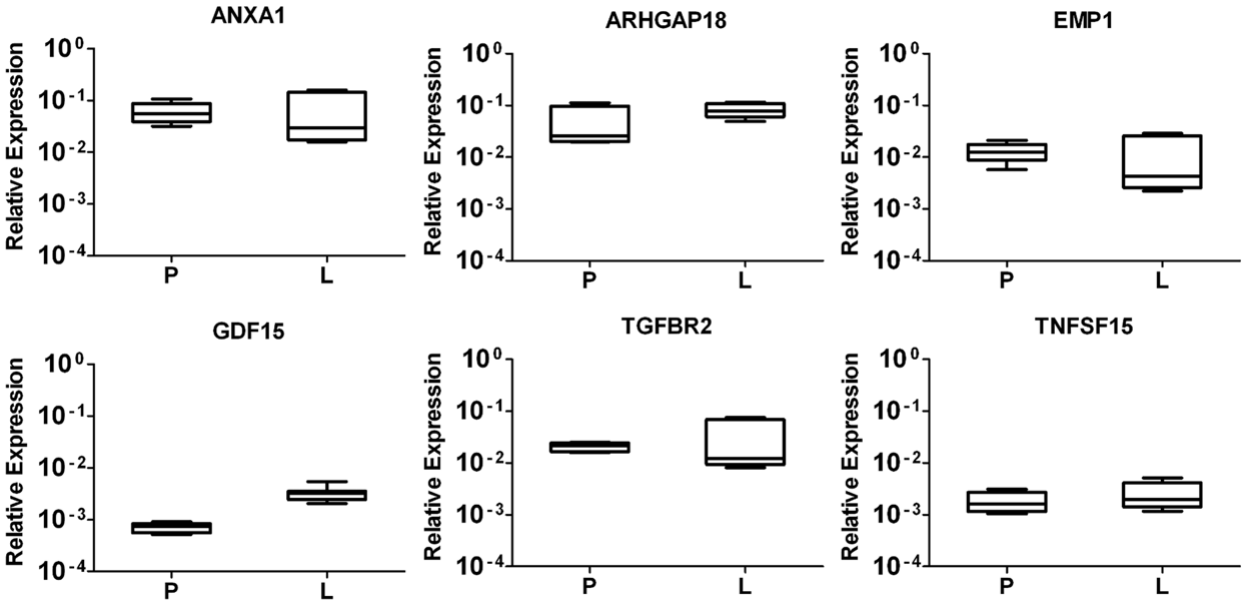
Gene expression of novel detected genes from laser capture microdissected tumour cells. Total RNA from microdissected tumour cells of three primary tumour (P) specimens and three liver metastases (L) were analysed by QRT-PCR. Results are plotted using the 2^−ΔΔCt^ method with *β-actin* expression (set to 1) from each individual sample as endogenous reference. Plotted results are means ± SD from triplicate wells.

**Figure 7:**
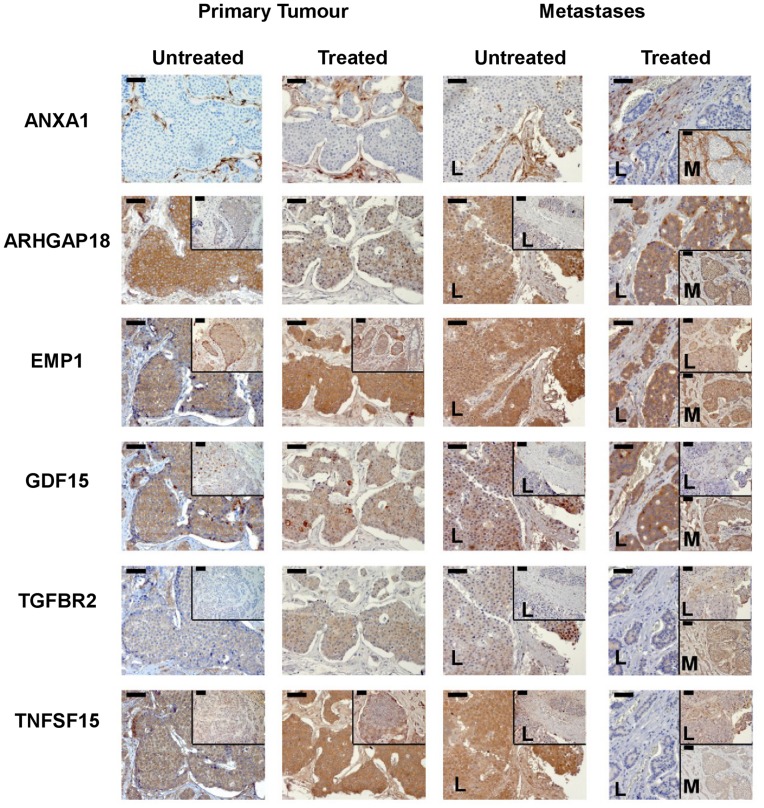
Immunohistochemistry of ANXA1, ARHGAP18, EMP1, GDF15, TGFBR2 and TNFSF15. Formalin-fixed paraffin-embedded tissue sections from 12 SI-NET patients at different stage of disease were used for the staining by using commercial antibodies. Three untreated primary tumours, 3 treated primary tumours, 3 untreated liver metastases, 2 treated liver metastases (L) and 1 treated mesentery metastasis (M). The results show a variety of specific pattern of expression of the different proteins, which are described in the Result paragraph. However, they clearly show the presence of 5 of the 6 selected encoded proteins in the tumor cells of SI-NET slides. Bar = 50 µm.

### Octreotide Clearly Reduces the Proliferation Rate of CNDT2.5 Cells

The CNDT2.5 cell line was used as an *in vitro* model to investigate the mechanisms whereby octreotide may alter neuroendocrine cell biology. Untreated and octreotide-treated CNDT2.5 cells were always cultured for the same time period in the different experiments. To determine the effect of octreotide on CNDT2.5 cell growth, we measured cell proliferation (metabolic activity of viable cells) in the absence or presence of 1 µM octreotide at 1 week, 4, 10 and 16 months using the commercial WST-1 assay. Octreotide reduces the growth of CNDT2.5 cells as shown in Figure 2. Briefly, 1 week of treatment produced a 15% cell growth reduction; 4 months, 26%; 10 months, 46%; and 16 months, 49% of growth reduction. In addition, the growth capacity was rapidly restored after octreotide withdrawal (Supporting Figure S1).

### Transcription Analysis of CNDT2.5 Cells in the Absence or Presence of Octreotide

To uncover the mechanism of action behind the reduction of cell growth rate induced by octreotide, CNDT2.5 cells were cultured for 10 and 16 months in the absence or presence of 1 µM octreotide. Collected total RNA was profiled using Affymetrix microarrays and raw data were deposited on NCBI’s GEO (GSE24358) and EBI’s Array-Express database (E-MTAB-388). Microarray analysis did not detect a high-fold change in the *SSTR* expression (Supporting Table S1). However, marked changes in expression of a number of genes in response to octreotide treatment were detected by the arrays. Twenty-five genes were reported as significantly informative by FARMS algorithm and were commonly regulated more than two fold at 10 and 16 months. The genes were then clustered according to Euclidian distance (Data not shown). We restricted our interest to 25 regulated genes after functional analysis and their gene expression is shown in Figure 3. Moreover, 25 genes were classified using gene ontology MAS 3 (Table 1). Then six genes, which are involved in proliferation/apoptosis and cell signaling, were selected for further validation after gene ontology and bioinformatics analysis. They are shown in bold in Table 1.

### QRT-PCR and Protein Analyses of Octreotide-treated CNDT2.5 Cells Confirm the Upregulation of Six Selected Genes

QRT-PCR analysis was extended to verify differences in transcript levels of *ANXA1*, *ARHGAP18*, *EMP1*, *GDF15*, *TGFBR2* and *TNFSF15* between CNDT2.5 cells cultured in the absence or presence of octreotide. Thus, *ANXA1*, *ARHGAP18*, *EMP1*, *GDF15*, *TGFBR2* and *TNFSF15* showed a clear accumulation of transcripts in response to octreotide treatment (Figure 4). To further verify the findings from the microarray analysis, we assessed the protein levels of SSTR 1-5 and the selected genes of interest using western blot analysis. Western blot analysis detected slight increases in the levels of SSTR 1, 2 and 3 proteins although not in SSTR5 (Supporting Figure S2) in line with the small increased expression of *SSTRs* from the microarray analysis. In addition, the five proteins (ANXA1, ARHGAP18, GDF15, TGFBR2 and TNFSF15) were markedly increased after 10 and 16 months octreotide treatment as shown in Figure 5A. In contrast, we were unable to detect EMP1, which in fact has never been detected in CNDT2.5 cells. The fold changes between untreated and treated cells are presented in a table for clarity in Figure 5B.

### Octreotide-upregulated Transcripts and Encoded Proteins of CNDT2.5 Cells and SI-NET Tumour Tissue at Different Stage of Disease

To better understand the octreotide-regulated genes, which were identified by microarray analyses and QRT-PCR of CNDT2.5 cells, we next analysed tumour tissue. We first verified the RNA expression of *ANXA1, ARHGAP18, EMP1, GDF15, TGFBR2* and *TNFSF15* using laser capture microdissected (LCM) tumour cells from 6 frozen blocks as illustrated in Table 2. Transcript expression in both primary tumours and liver metastases is evident for all the six genes (Figure 6). We then investigated protein expression using commercial antibodies for immunohistochemistry. ANXA1, ARHGAP18, EMP1, GDF15, TGFBR2 and TNFSF15 protein expression was analysed on tissue sections from 12 patients as illustrated in Table 2. The proteins show a variety of different expression pattern and the results are shown in Figure 7. ANXA 1 is the only protein stained in stroma cells and not in tumour cells, while the other five stains specifically either untreated tumour cells or treated ones at different stage of disease. In addition, the inserts of each panel in the figure show a weaker staining of the proteins, except for TNFSF15. A clear summary of the results is shown in Table 3.

## Discussion

NET patients require medical management that aims to relieve symptoms and suppress tumour growth and spread. Somatostatin and its stable analogues (octreotide, lanreotide and vapreotide) exert an antiproliferative effect on various normal and cancerous cells both *in vitro* and *in vivo*. Medical approaches in the management of NETs are limited and SSAs are a valuable resource to alleviate their symptoms resulting from the secretion of hormones or vasoactive peptides into the systemic circulation [6]. Moreover, the antitumoral effects of SSAs, recently demonstrated in SI-NETs [13] have prompted considerable interest in their use against this kind of rare tumours.

The main aim of our study was to identify potential novel genes, rather than the *SSTRs,* behind the effect of octreotide on NET cells. We used CNDT2.5 cells, as an *in vitro* model to investigate how octreotide may alter neuroendocrine cell biology in regard to tumour cells that express low levels of *SSTRs*. We studied whether and how cell growth control and differentiation can occur to the cells under daily octreotide treatment for up to 16 months, by applying long-term treatment with high concentration of octreotide. Despite the long treatment, CNDT2.5 cells maintained sensitivity to the drug. Moreover, they did not show susceptibility to apoptosis while the growth rate was steadily reduced over time. The growth capacity was rapidly restored after octreotide withdrawal (Supporting Figure S1). Furthermore, to better clarify the mechanism of action behind the reduction of cell growth induced by octreotide on CNDT2.5 cells, we gene profiled the cells to investigate variations in transcript levels after long-term octreotide treatment. By using microarray expression analysis, the expression levels of several hundred genes can be detected simultaneously and bioinformatics and gene ontology analyses suggested that 25 genes are differentially regulated by octreotide treatment. We choose 6 genes in the list, which were previously unrelated to octreotide signaling, excluding others for three main reasons. First, the genes were selected according to the biological functions inherent to proliferation/apoptosis and cell signaling by gene ontology. Second, the transcripts are not alternatively spliced to a large degree, which makes the preparation of QRT-PCR primers ideal. Third, commercial antibodies were available to extend the investigation to protein expression both in CNDT2.5 cellular total lysates and tissue specimens. Thus, these genes which are associated to proliferation/apoptosis and cell signaling may be involved in mediating octreotide-induced CNDT2.5 cell growth reduction.

Our results show that different times of octreotide treatment triggered a progressive increase in transcription of the all selected genes (*ANXA1*, *ARHGAP 18*, *EMP1*, *GDF15*, *TGFBR2* and *TNFSF15*), without altering the expression levels of SSTRs (Data not shown) in CNDT2.5 cells. Moreover, we confirmed induced upregulation by octreotide for most of these genes at the protein level. Thus, we suggest that octreotide may be effective in controlling growth of CNDT2.5 cells for a long time without triggering any resistance during a long treatment. Furthermore, results indicate that even cells or tissues expressing low levels of SSTRs, such as those tested here, show significant responses to octreotide.

In this scenario, our present results reveal a potential role for six select genes, *ANXA1, ARHGAP18, EMP1, GDF15, TGFBR2* and *TNFSF15*, which have not been explored hitherto in this field, and therefore deserve further investigation. *ANXA1, EMP1, TGFBR2* and *TNFSF15* have major roles in biological processes such as cell proliferation and apoptosis [30], [31], [32], [33], whereas *ARHGAP18* and *GDF15* have major roles in signal transduction [34], [35]. Moreover, both *ARHGAP18* and *EMP1* might work as tumour suppressors [36], [37].

Briefly, ANXA1 is a protein involved in adhesion, proliferation, apoptosis, migration, growth and differentiation [38]. It has also been proposed that ANXA1 expression may correlate with the tumorigenesis, of thyroid and gastric cancer, which emphasizes the importance of ANXA1 in different kinds of cancers [30], [39]. ARHGAP18 encodes rho GTPase-activating protein 18, which belongs to the RhoGAP family and functions as GTPase activator for the Rho-type GTPases. Notably, the RhoGAP family includes several tumour suppressors [40] and the role of Rho GTPases in diverse NETs signaling pathway has been established [35]. EMP1 encodes epithelial membrane protein 1, a multi-pass membrane protein that plays a role as a biomarker of gefitinib clinical resistance [41], [42]. EMP1 is involved in the EGFR signaling pathway with an important role in cell proliferation and epithelial cell differentiation and it is considered important in metastatic colorectal cancer [43]. GDF15 encodes growth/differentiation factor 15, a member of the transforming growth factor beta (TGF beta) superfamily that regulates tissue differentiation [44]. In addition, this protein has a variety of roles in growth, apoptosis, survival, proliferation and signaling [45]. TGFBR2 encodes TGF-beta II receptor, a member of the Ser/Thr protein kinase family, which is part of the TGFB receptor superfamily [46]. The protein is a single-pass type I membrane protein, receptor for TGF-beta, an important regulator of cell proliferation, differentiation and extracellular matrix production. Mutations in this gene have been associated with the development of various types of tumours [47]. Explicitly it has a defined role in colon cancer and a combination of inactivation of the TGF-3 signaling pathway and expression of oncogenic Kras leads to formation of invasive intestinal neoplasms through a beta-catenin-independent pathway [48]. Last, TNFSF15 encodes a cytokine, which belongs to the tumor necrosis factor (TNF) ligand family and its second common ID is VEGI, vascular endothelial cell growth inhibitor. This protein is abundantly expressed in endothelial cells and never in B or T cells. Protein expression is inducible by TNF and IL-1 alpha. This cytokine is a ligand for receptor TNFRSF25 and decoy receptor TNFRSF21/DR6. It can activate NF-kappaB and MAP kinases, and acts as an autocrine factor to induce apoptosis in endothelial cells. This cytokine is also found to inhibit endothelial cell proliferation, and thus may function as an angiogenesis inhibitor. However, the complex function in diseases and health is summarized in a 2011 article, which considers its pivotal role in cancer-tumor-immonology [49].

However, we were not able to obtain unequivocal evidence that the six proposed genes have a major role on octreotide direct effects. Indeed, it is very well known that this drug may have a capacity to control changes of some structural properties of the cells via secondary effects. In addition, the *in vitro* antiangiogenic effects of somatostatin and its analogues have been previously shown by studies on melanoma cells, which expressed one or more SSTR and were treated by using somatostatin or SSAs [20], [50].

As far as we know, we are the first group proposing that octreotide may signal its effects through *SSTRs* by activating a set of genes that have not previously been associated to the conventional octreotide signaling pathway. It is today not known how downstream events lead to differential expression of the six described genes. Indeed, we have been treating our *in vitro* model up to 16 months, showing that EMP1, GDF15 and TGFBR2 are modulated after 10 months of treatment. This potentially suggests that they are not involved at the earlier time point when octreotide is able to inhibit cell proliferation. Thus, this may imply that EMP1, GDF15 and TGFBR2 might be either involved in octreotide indirect effects or being pivotal in opposing cell growth control via a downstream network. However, the six genes represent new, valuable tools for the identification of novel potential biomarkers or therapeutically relevant targets. Hence, the whole-transcript expression analysis of CNDT2.5 cells offers a powerful and informative resource for detecting variation in gene expression between non-treated and long-term octreotide-treated CNDT2.5 cells. Most importantly, extension of our analysis to primary SI-NET and liver metastases showed that these genes encode proteins in tumour tissues. Although it is difficult to access to the right number of tumour specimens to translate the results *in vitro* to clinical results, we strongly believe that the novel potential mechanisms used by octreotide to control NET biology should be investigated.

Thus, octreotide may signal through alternative mechanisms that require expression of *SSTRs* in combination with different genes, which may activate a potential novel framework, which is not fully understood. However, our transcriptomic analysis detected 6 novel genes, which may encode proteins able to cross talk. Thus, *SSTRs, ANXA1, ARHGAP18, EMP1, GDF15, TGFBR2* and *TNFSF15* genes and encoded proteins may have a potential role in controlling cell growth and differentiation in human NET cells by octreotide. Moreover, investigating this new potential framework help our understanding about why patients get relief from SSA therapy when they do not initially overexpress high amount of *SSTRs* [51], [52]. The newly detected proteins control many different cellular mechanisms. Thus, further analyses are necessary to fully understand the new mechanisms of octreotide to control NET cells growth and differentiation. Ideally, an animal model may clarify better our hypothesis.

## Methods

### Ethics Statement

This study was conducted according to the principles expressed in the Declaration of Helsinki. Moreover, this study was approved by the regional Ethical Committee at the Clinic of Endocrine Oncology, Uppsala University Hospital, Sweden. All the participants provided written consent for studying the tissue samples and eventually to publish new obtained scientific data. The study was performed in accordance with the regional Ethical Committee at the Clinic of Endocrine Oncology, Uppsala University Hospital, Sweden (approval number: Dnr 2011/426).

### Human NET Cell Lines and Media

CNDT2.5, KRJ-1 and QGP-1 were gifts from Prof. L.M. Ellis, MD Anderson Cancer Center, Houston, TX, USA, Prof. I.M. Modlin, Yale University, New Haven, CT, USA and Prof. B. Ericsson, Uppsala University Hospital, Uppsala, Sweden respectively. Moreover, these cells were previously used for scientific studies, which have been published as You can see in the follow references [27], [28], [29]. The human lung carcinoids cell lines NCI-H720 and NCI-H727 were from ATCC (LGC Promochem, Sweden). The cells were cultured at 37°C and 5% CO_2_-humidified atmosphere in culture media as previously reported [27], [28], [29], [53].

### Tissue Samples

The tissue samples included in the study have a histopathologically confirmed diagnosis of SI-NETs. Snap-frozen specimens from 6 patients were used to isolate total RNA from laser capture microdissected (LCM) tumour cells. Moreover, formalin fixed paraffin-embedded tissues from 12 patients were used for immunohistochemistry (IHC). Patients’ information is summarized in Table 2. Permission to collect tumour specimens was approved by the regional Ethical Committee at the Uppsala University Hospital (Dnr 2011/426).

### Laser Capture Microdissection of NET Cells

Snap-frozen specimens from three untreated primary tumours and three SSA+IFN treated liver metastases were cut in 8-µm sections by a microtome cryostat (Leica, Wetzlar, Germany) and adhered to polyethylene-naphtalate membrane frame slides (Applied Biosystems, Life Technologies, Carlsbad, California, USA). Primary tumour cells and liver metastatic cells were isolated by Arcturus^XT^ Microdissection system (Applied Biosystems) according to the manufacturer’s instructions.

### RNA Extraction From Cell Lines and Laser Capture Microdissected Tumour Cells

Total RNA was isolated from five human neuroendocrine cancer cell lines with PARIS Kit (Applied Biosystems) according to the manufacturer’s instructions. RNAqueous Micro Kit (Applied Biosystems) was used to prepare total RNA from laser capture microdissected tumour cells. RNA quantity and quality was always verified by using the RNA 6000 Nano Kit/RNA 6000 Pico Kit and the Agilent 2100 Bioanalyzer (Agilent technologies, Waldbronn, Germany).

### Microarray Data Analysis and Data Mining

About one microgram of total RNA per each cell line described above was sent to the Uppsala Array Platform, Uppsala University Hospital, Uppsala, Sweden. Total RNA was hybridized onto the Affymetrix Human Gene 1.0 ST Array (Affymetrix, Santa Clara, CA, USA) and processed according to Affymetrix technical protocols. Scanned images of microarray chips were analysed by the GeneChip Operating Software (Affymetrix). Untreated CNDT2.5 cells and treated CNDT2.5 cells were always cultured at the same time. We profiled gene expression of untreated human neuroendocrine cancer cells CNDT2.5 and 1 µM octreotide treated CNDT2.5 cells after 10 and 16 months of culture. The raw data were normalized using RMA algorithm. This analysis was performed with the MeV software (www.tm4.org) [54]. Before comparing the effect of long time (10 months and 16 months) octreotide treatment on CNDT2.5 cells gene expression, microarray raw data were normalized using FARMS normalization algorithm as implemented in XPS, package (R/Bioconductor: www.bioconductor.org). Significantly marked genes as informative with a present call by I/NI-calls algorithm were kept for further fold change analysis between treated and untreated cells. A hierarchical clustering algorithm was applied to group genes and samples according to similarities in expression. Genes differentially expressed were clustered using Euclidian distance with average linkage clustering (genes and samples). Gene function based on gene ontology analysis was performed by using IHOP - Gene Model (www.ihop-net.org/UniPub/iHOP/) and MAS 3(http://bioinfo.capitalbio.com/mas3/).

### Cell Proliferation Assay

Spectrophotometric quantification of cell proliferation was measured by using the metabolic proliferation reagent WST-1 (Roche Applied Science, Mannheim, Germany) according to the manufacturer’s instructions. The principle of this assay relies on the cleavage of the stable tetrazolium salt WST-1 to a soluble formazan by a complex enzymatic cellular mechanism. This bioreduction mainly depends on the glycolytic production of NAD(P)H in viable cells. Thus, the amount of formazan dye formed directly correlates to the number of metabolically active cells in the culture. CNDT2.5 cellswere cultured in the absence and presence of otreotide for 1 week, 4, 10 and 16 months. We first performed WST-1 assay for each time point. Then we repeated the assay two times using cells frozen at 1 week, 4, 10 and 16 months. Octreotide was used at the concentration of 1 µM and cells were seeded in 96-well plates 100 µL/well at a density of 1.2×10^3^ cells per well. Moreover, cells were cultured in the presence or absence of 1 µM octreotide for 1 week to evaluate cell proliferation. Cells were then incubated for 1 h at 37°C in a 5% CO_2_ atmosphere in the presence of metabolic reagent WST-1 (10 µL/well). The absorbance of the samples against a background control as blank (media) was measured at 450 nm by using a Multiskan Ascent microplate (ELISA) reader (Thermo Scientific, Rockford, IL, USA). Cell proliferation was calculated as a percentage of untreated CNDT2.5 cells. WST-1 data were plotted using the results from three independent wells.

### QRT-PCR

About 1 µg of total RNA per sample was converted to cDNA with iScript cDNA synthesis Kit (Bio-Rad, Hercules, CA, USA). Briefly, to estimate the starting copy number of cDNA, sample signal was compared with that generated with a specific standard curve containing 1, 10^1^, 10^2^, 10^3^, 10^4^, 10^5^ and 10^6^ copies of synthetic cDNA template for each transcript of interest run on the same plate. QRT-PCR verified mRNA levels of all SSTR-subtypes have been recently reported [55]. Results are expressed as copies of *SSTR* per copies of *β-actin*. QRT-PCR primers and amplicons are described in Supporting Table S2. *ANXA1*, *ARHGAP18*, *EMP1*, *GDF15*, *TGFBR2* and *TNFSF15* were measured using Stratagene Mx3005P real time PCR System (Agilent technologies) and brilliant SYBR Green QPCR Master Mix (Agilent technologies). The data were evaluated by the 2^−ΔΔCT^ method [56] using the mRNA level of *β-actin* (set to 1). QRT-PCR primers and amplicons are described in Supporting Table S3.

### Western Blot Analysis

CNDT2.5 cells were cultured in the absence or presence of octreotide 1 µM and total protein lysates were collected at 1 week, 4, 10 and 16 months. Whole-cell protein lysates were extracted by using radio-immunoprecipitation assay buffer. Cells were at 70% to 80% confluence as previously described [27]. Protein concentrations were determined using Coomassie-Plus Better BradFord Assay (Thermo Scientific). Aliquots of 70 µg were resolved by precast any kD Mini-PROTEAn TGX gels (Bio-Rad) and transferred to 0.45-µm nitrocellulose membranes (Bio-Rad). Benchmark pre-stained protein ladder (Invitrogen, Carlsbad, California, USA) was used to calculate the apparent size of proteins. The membranes were blocked with Western Blocking Reagent (Roche Applied Science) overnight and then blotted with the primary antibody overnight at 4°C. Then they were washed and incubated with the horseradish peroxides-conjugated (HRP) secondary antibody for 1 h at room temperature and washed again. The blots were visualized with Lumi-Light Western Blotting Substrate (Roche Applied Science). Monoclonal mouse anti-human ANXA1 (1∶5000, BD-Transduction Laboratories, Franklin Lakes, NJ, USA), polyclonal rabbit anti-human ARHGAP18 (1∶250, Abgent, San Diego, CA, USA), polyclonal mouse anti-human EMP1 (1∶250, Abnova, Taipei, Taiwan), polyclonal rabbit anti-human GDF15 (1∶250, Atlas Antibodies, Stockholm, Sweden), polyclonal goat anti-human TGFBR2 (1∶250, Santa Cruz Biotechnology, Santa Cruz, CA, USA), polyclonal goat anti-human TNFSF15 (1∶500, R&D Systems, Minneapolis, MN, USA), polyclonal rabbit anti-human SSTR1 (1∶5000, Santa Cruz Biotechnology), polyclonal rabbit anti-human SSTR2 (1∶4000, Thermo Scientific), polyclonal rabbit anti-human SSTR3 and SSTR5 (1∶4000 and 1∶5000, gifts from Frank Leu [19], polyclonal goat anti-human β-actin and HRP donkey anti-goat (1∶5000, Santa Cruz Biotechnology) and HRP anti-mouse and anti-rabbit (1∶5000, Amersham Biosciences, Buckinghamshire, England) antibodies were used to detect the different proteins.

### Immunohistochemistry

We selected paraffin-embedded tissue slides from 12 patients (Table 2) to investigate the differentially expressed markers. We used anti-human ANXA1 (1∶1000), anti-human ARHGAP18 (1∶500), anti-EMP1 (1∶500), anti-human GDF15 (1∶100), a different polyclonal rabbit anti-human TGFBR2 (1∶500) from Abbiotec, San Diego, CA, USA and a different polyclonal rabbit anti-human TNFSF15 (1∶1000), from Acris Antibodies, Herford, Germany. The staining was performed as described elsewhere [57]. Results were evaluated using Axiophot light microscope and AxioVision Rel.4.5 software (Carl Zeiss AG, Oberkochen, Germany).

### Statistical Analysis

Results are shown as mean ± SD. All the experiments were performed at least in triplicate. The statistical significance of the difference between two groups was evaluated by two-tailed Student’s *t*-test or Two-Way ANOVA followed by Bonferroni test using GraphPad Prism 5 (Graph Pad, Software, La Jolla CA, USA); *p* value <0.05 is considered significant.

## Supporting Information

Figure S1 **CNDT2.5 cells growth in the presence of 1 µM octreotide for 15 or 16 weeks.** Cells were cultured in the absence or presence of 1 µM octreotide. Cell proliferation rate was converted to a percentage of the mean value relative to the untreated CNDT2.5 cells, set to 100% and results represent means ± SD from triplicate wells. Significance was calculated by student *t*-test, comparing with untreated CNDT2.5 cells. * *p*<0.05, ** *p*<0.001.
(TIF)

Figure S2 **SSTR1, SSTR2, SSTR3 and SSTR5 protein expression.** CNDT2.5 cells were cultured in the absence or presences of 1 µM octreotide (oct). They were collected at 1 week (wk) and 16 months (mo) for preparing total lysates and performing western blot analysis. β-actin was used as endogenous control. Western blot results are shown on the left and the table shows the protein fold change on the right.
(TIF)

Table S1 Microarray data of somatostatin receptors 1–5 on CNDT 2.5 cells.
(DOC)

Table S2 Primer pairs of SSTRs used for QRT-PCR analysis.
(DOC)

Table S3 Primer pairs of selected genes used for QRT-PCR analysis.
(DOC)
