# Supplementary figures and images for: The Somatostatin Analogue Octreotide Inhibits Growth of Small Intestine Neuroendocrine Tumour Cells
Source: PLoS One. 2012 Oct 31;7(10):e48411. doi: 10.1371/journal.pone.0048411 (PMC3485222; doi:10.1371/journal.pone.0048411)

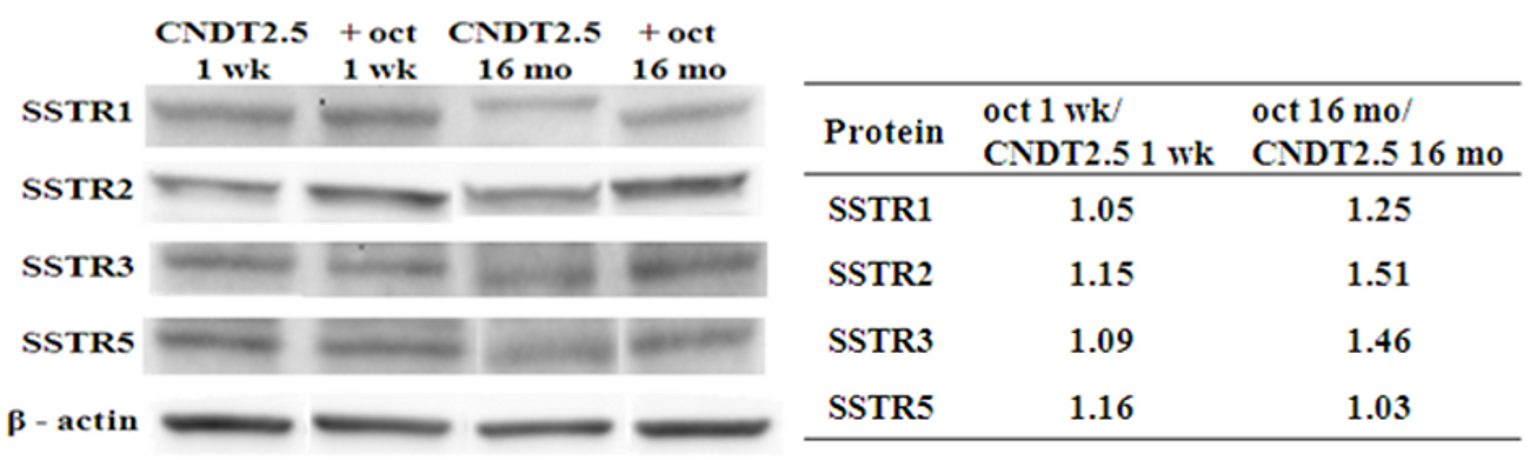

Supplement: Figure S2 — SSTR1, SSTR2, SSTR3 and SSTR5 protein expression. CNDT2.5 cells were cultured in the absence or presences of 1 µM octreotide (oct). They were collected at 1 week (wk) and 16 months (mo) for preparing total lysates and performing western blot analysis. β-actin was used as endogenous control. Western blot results are shown on the left and the table shows the protein fold change on the right. (TIF) [file pone.0048411.s002.tif]
