# Supplementary material for: The Somatostatin Analogue Octreotide Inhibits Growth of Small Intestine Neuroendocrine Tumour Cells
Source: PLoS One. 2012 Oct 31;7(10):e48411. doi: 10.1371/journal.pone.0048411 (PMC3485222; doi:10.1371/journal.pone.0048411)
Supplement: Table S1 — Microarray data of somatostatin receptors 1–5 on CNDT 2.5 cells. (DOC) [file pone.0048411.s003.doc]

**Supporting Table S1.** Microarray data of somatostatin

receptors 1-5 on CNDT 2.5 cells.

| **Gene Symbol** | **10 mo** | **oct 10 mo** | **16 mo** | **oct 16 mo** |
| --- | --- | --- | --- | --- |
| SSTR1 | 4.6 | 5.2 | 5.2 | 5.2 |
| SSTR2 | 5.7 | 5.6 | 5.7 | 5.7 |
| SSTR3 | 6.3 | 6.2 | 6.4 | 6.3 |
| SSTR4 | 4.4 | 5.5 | 5.9 | 5.6 |
| SSTR5 | 5.9 | 5.2 | 5.9 | 6.0 |

Month (mo); Octreotide (oct)
