# Supplementary material for: The Somatostatin Analogue Octreotide Inhibits Growth of Small Intestine Neuroendocrine Tumour Cells
Source: PLoS One. 2012 Oct 31;7(10):e48411. doi: 10.1371/journal.pone.0048411 (PMC3485222; doi:10.1371/journal.pone.0048411)
Supplement: Table S2 — Primer pairs of SSTRs used for QRT-PCR analysis. (DOC) [file pone.0048411.s004.doc]

**Supporting Table S2.** Primer pairs of *SSTRs* used for QRT-PCR analysis.

| **Symbol** | **Description** | **Primer Sequences** | **Product (bp)** |
| --- | --- | --- | --- |
| *SSTR1* | somatostatin receptor 1 | F: 5'-CACATTTCTCATGGGCTTCCT-3' | 165 |
|  |  | R: 5'-ACAAACACCATCACCACCATC-3' |  |
| *SSTR2* | somatostatin receptor 2 | F: 5'-GGCATGTTTGACTTTGTGGTG-3' | 185 |
|  |  | R: 5'-GTCTCATTCAGCCGGGATTT-3' |  |
| *SSTR3* | somatostatin receptor 3 | F: 5'-TGCCTTCTTTGGGCTCTACTT-3' | 190 |
|  |  | R: 5'-ATCCTCCTCCTCAGTCTTCTCC-3' |  |
| *SSTR4* | somatostatin receptor 4 | F: 5'-CGTGGTCGTCTTTGTGCTCT-3' | 174 |
|  |  | R: 5'-AAGGATCGGCGGAAGTTGT-3' |  |
| *SSTR5* | somatostatin receptor 5 | F: 5'-CTGGTGTTTGCGGGATGTT-3' | 183 |
|  |  | R: 5'-GAAGCTCTGGCGGAAGTTGT-3' |  |
| *ACTB* | actin, beta (β-actin) | F: 5'-ACTCTTCCAGCCTTCCTTCCT-3' | 176 |
|  |  | R: 5'-CAGTGATCTCCTTCTGCATCCT-3' |  |
