# Supplementary material for: The Somatostatin Analogue Octreotide Inhibits Growth of Small Intestine Neuroendocrine Tumour Cells
Source: PLoS One. 2012 Oct 31;7(10):e48411. doi: 10.1371/journal.pone.0048411 (PMC3485222; doi:10.1371/journal.pone.0048411)
Supplement: Table S3 — Primer pairs of selected genes used for QRT-PCR analysis. (DOC) [file pone.0048411.s005.doc]

**Supporting Table S3.** Primer pairs of selected genes used for QRT-PCR analysis.

| **Symbol** | **Description** | **Primer Sequences** | **Product (bp)** |
| --- | --- | --- | --- |
| *ANXA1* | annexin A1 | F: 5'-GCTATCCACAACTTCGCAGA-3' | 119 |
|  |  | R: 5'-CACGATAGCTGTGAGGCATT-3' |  |
| *ARHGAP18* | Rho GTPase protein 18 | F: 5'-GCCTAATTGAGCTGACTGC-3' | 153 |
|  |  | R: 5'-TCGCATTCCTGGTACTTTCC-3' |  |
| *EMP1* | epithelial membrane 1 | F: 5'-AACTCTTGTGGTACCTAGTCAGATGGTA-3' | 113 |
|  |  | R: 5'-GCAAAGCAATGCCTGCTTAAC-3' |  |
| *GDF15* | growth differentiation 15 | F: 5'-TGCGGAAACGCTACGAGGAC-3' | 210 |
|  |  | R: 5'-GGAACAGAGCCCGGTGAAGG-3' |  |
| *TGFBR2* | transforming growth beta | F: 5'-CCATGTCTCACAGCCAGCTA-3' | 155 |
|  |  | R: 5'-CCAGGAGAAATAAGGGCACA-3' |  |
| *TNFSF15* | tumor necrosis factor 15 | F: 5'-GCAGGACTCACCACATACC-3' | 147 |
|  |  | R: 5'-CCTTGGCTTATCTCCGTCTG-3' |  |
| *ACTB* | actin, beta (β-actin) | F: 5'-CGCGAGAAGATGACCCAGAT-3' | 71 |
|  |  | R: 5'-ACAGCCTGGATAGCAACGTACA-3' |  |
